# Supplementary material for: Economic segregation is associated with reduced concerns about economic inequality
Source: Nat Commun. 2024 Jul 5;15:5655. doi: 10.1038/s41467-024-49778-w (PMC11226429; doi:10.1038/s41467-024-49778-w)
Supplement: Supplementary file 1 — Supplementary Information [file 41467_2024_49778_MOESM1_ESM.pdf]

Supplemental Materials for

Economic segregation is associated with reduced concerns about economic inequality

Shai Davidai, Columbia University

Daniela Goya-Tocchetto, University at Buffalo, SUNY

M. Asher Lawson, INSEAD

Supplementary Note 1: Exploratory analyses of inequality X segregation interaction effect for Study 1

1. Table S1. Coefficients from regressing attitudes about economic inequality on independent variables and their two-way interaction.
2. Figure S1. The specification curve for the interactive effect of income segregation and income inequality on people's support for redistribution

Supplementary Note 2: An alternative approach to direct geographical aggregation in Study 1

a) Table S2. Average effect sizes, % negative effects, and % positive effects across two approaches to aggregation (Direct aggregation vs. Population weighting)

Supplementary Note 1: Exploratory analyses of inequality X segregation interaction effect for Study 1

In addition to the results reported in the manuscript, we report the results of an exploratory analysis estimating the interactive effect of economic segregation and economic inequality (as measured by the Gini coefficient) on people's concern about inequality. As shown in Table S1, we found a significant positive interaction between segregation and the Gini coefficient. While economic segregation negatively predicted concern about inequality when it was relatively low, the relationship between segregation and attitudes about inequality was attenuated at higher levels of inequality, suggesting that at extreme levels of inequality segregation may no longer dampen people's concern about it. However, as shown in Model 2, this interactive effect was no longer significant when controlling for the additional variables in the analysis. Moreover, as shown in Figure S1, a robustness analysis of the interaction effect across various model specifications revealed that it was positively significant for only 28.0% of the specifications (and negatively significant for 15.4% of the specifications), suggesting that the interaction effect between economic segregation and economic inequality was not especially robust. Thus, we did not find consistent statistically significant evidence that the level of inequality moderates the effect of segregation.

Table S1. Coefficients from regressing attitudes about economic inequality on independent variables and their two-way interaction.

	Model 1	Model 2
Economic segregation (ES)		
Coefficient	-0.100	-0.272
CI	[-0.131, -0.069]	[-0.400, -0.144]
p	< .001	< .001
Gini coefficient (GC)		
Coefficient	0.029	0.087
CI	[0.010, 0.048]	[0.032, 0.142]
p	0.003	0.002
Population		
Coefficient		0.003
CI		[-0.070, 0.076]
p		0.937
Income per capita		
Coefficient		0.103
CI		[-0.002, 0.208]
p		0.055
Proportion of Black residents		
Coefficient		-0.056
CI		[-0.124, 0.012]
p		0.106
Racial segregation		
Coefficient		0.109
CI		[0.016, 0.201]
p		0.022
Proportion Democrats		
Coefficient		-0.012
CI		[-0.071, 0.048]
p		0.704
ES x GC		
Coefficient	0.059	0.038
CI	[0.046, 0.073]	[-0.038, 0.115]
p	< .001	0.327
Constant		
Coefficient	-0.031	-0.020
CI	[-0.156, 0.094]	[-0.162, 0.121]
p	0.627	0.778

Results from two-sided multiple linear regression analyses predicting attitudes about inequality from Economic segregation (ES), Gini coefficient (GC), Population, Income per capita, Proportion of Black residents, Racial segregation, Proportion of Democrat vote, and the interaction term between ES and GC.

Figure S1. The specification curve for the interactive effect of income segregation and income inequality on people's support for redistribution.

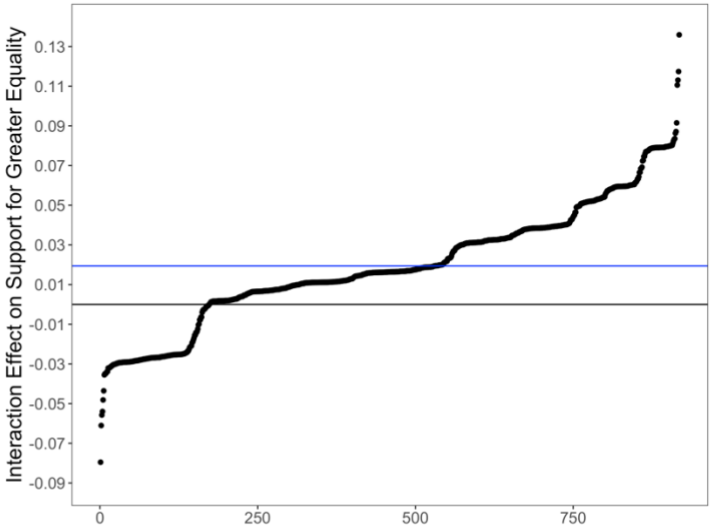

Note. The horizontal blue line denotes the average effect size across specifications. The x-axis denotes each specification number in the model. The horizontal black line denotes a null effect (i.e., 0).

Supplementary Note 2: An alternative approach to direct geographical aggregation in Study 1

In the process of conducting the analyses for Study 1, we ran an exploratory analysis using an alternative method of aggregating data from Commuter Zones level to State-level. In the primary analyses (reported in the main text), we used the most straightforward approach for data aggregation, averaging Commuter Zone data within a state to form a single State-level estimate (i.e., direct aggregation approach). Table S2 compares the results of this direct aggregation approach to an alternative approach, weighing Commuter Zones by their population (i.e., population weighting approach). Although aggregating CZs using population weighting attenuated the size of the observed effects (main effect of segregation, interaction effect of segregation and inequality,), it did not substantially impact their significance. Importantly, the alternative population weighting approach had essentially no effect on the sign nor significance of the main effect of segregation on concerns about inequality. Thus, the results across the two different approaches indicated a robust negative effect of economic segregation on concerns about inequality and a more fragile interaction between segregation and inequality.

Although the two approaches yield similar results, we have reason to believe that the more straightforward direct aggregation approach is more theoretically suitable for investigating the particular question at hand. Of note, the two methods of aggregation vary in their weighing of urban and rural areas, with the direct aggregation approach underweighting more populous urban areas and the population weighting approach overweighting such areas. Thus, while the direction aggregation approach is most suitable for estimating an effect within a randomly selected location, the population weighing approach is more suitable for estimating an effect among a randomly selected person. Given that our independent variable is a geographical measure of

segregation, directly aggregating across geographical regions is thus more suitable for the current analyses. Moreover, this method of aggregation allows our analyses to focus on a state as a whole rather than giving undue attention to urban areas within it.

Table S2. Average effect sizes, % negative effects, and % positive effects across two approaches to aggregation (Direct aggregation vs. Population weighting)

	Main effect (Economic Segregation)		Interaction effect (Segregation X Inequality)	
	Direct aggregation	Population weighting	Direct aggregation	Population weighting
Average effect size	-0.150	-0.076	0.019	0.002
% Negative effects	100%	100%	18.85%	39.33%
% Negative and significant	98.15%	98.04%	15.36%	4.03%
% Positive effects	0%	0%	81.16%	60.68%
% Positive and significant	0%	0%	28.00%	13.18%
