# Supplementary material for: Economic segregation is associated with reduced concerns about economic inequality
Source: Nat Commun. 2024 Jul 5;15:5655. doi: 10.1038/s41467-024-49778-w (PMC11226429; doi:10.1038/s41467-024-49778-w)
Supplement: Supplementary file 3 — Reporting Summary [file 41467_2024_49778_MOESM3_ESM.pdf]

nature portfolio

Corresponding author(s): Shai Davidai

Last updated by author(s): Mar 22, 2024

Reporting Summary

Nature Portfolio wishes to improve the reproducibility of the work that we publish. This form provides structure for consistency and transparency in reporting. For further information on Nature Portfolio policies, see our Editorial Policies and the Editorial Policy Checklist.

Statistics

For all statistical analyses, confirm that the following items are present in the figure legend, table legend, main text, or Methods section.

n/a	Confirmed	
☐	☒	The exact sample size (n) for each experimental group/condition, given as a discrete number and unit of measurement
☐	☒	A statement on whether measurements were taken from distinct samples or whether the same sample was measured repeatedly
☐	☒	The statistical test(s) used AND whether they are one- or two-sided<br>*Only common tests should be described solely by name; describe more complex techniques in the Methods section.*
☐	☒	A description of all covariates tested
☐	☒	A description of any assumptions or corrections, such as tests of normality and adjustment for multiple comparisons
☐	☒	A full description of the statistical parameters including central tendency (e.g. means) or other basic estimates (e.g. regression coefficient) AND variation (e.g. standard deviation) or associated estimates of uncertainty (e.g. confidence intervals)
☐	☒	For null hypothesis testing, the test statistic (e.g. F, t, r) with confidence intervals, effect sizes, degrees of freedom and P value noted<br>*Give P values as exact values whenever suitable.*
☒	☐	For Bayesian analysis, information on the choice of priors and Markov chain Monte Carlo settings
☐	☒	For hierarchical and complex designs, identification of the appropriate level for tests and full reporting of outcomes
☐	☒	Estimates of effect sizes (e.g. Cohen's d, Pearson's r), indicating how they were calculated

Our web collection on statistics for biologists contains articles on many of the points above.

Software and code

Policy information about availability of computer code

Data collection	The experimental data was collected using Qualtrics XM.
Data analysis	The code for the analyses of Study 1 is available online through the Open Science Framework (10.17605/OSF.IO/U2T7D) and is also provided in the manuscript under the "Code Availability" statement

For manuscripts utilizing custom algorithms or software that are central to the research but not yet described in published literature, software must be made available to editors and reviewers. We strongly encourage code deposition in a community repository (e.g. GitHub). See the Nature Portfolio guidelines for submitting code & software for further information.

Data

Policy information about availability of data

All manuscripts must include a data availability statement. This statement should provide the following information, where applicable:

- Accession codes, unique identifiers, or web links for publicly available datasets
- A description of any restrictions on data availability
- For clinical datasets or third party data, please ensure that the statement adheres to our policy

The following Data Availability Statement is included in the manuscript:

The materials and datasets generated for Studies 2a-5b have been deposited in the Open Science Framework (OSF) repository and are accessible here: 10.17605/

OSF.IO/U2T7D. Study 1 used two publicly available datasets: the World Values Survey (www.worldvaluessurvey.org) and the Opportunity Insights dataset (including data from the 2000 U.S. census; https://rajchetty.com/wp-content/uploads/2021/04/land-of-opportunity.html-charsetutf-8).

Research involving human participants, their data, or biological material

Policy information about studies with human participants or human data. See also policy information about sex, gender (identity/presentation), and sexual orientation and race, ethnicity and racism.

Reporting on sex and gender	Gender based information was collected as self-reported and is described in the methods section in the manuscript. The reported findings were not moderated by gender.
Reporting on race, ethnicity, or other socially relevant groupings	Participants self-reported their ethnic identity. This variable was collected for mere demographic descriptors and was not used as a proxy for other socially constructed variables.
Population characteristics	See below.
Recruitment	Participants were recruited from Amazon's Mechanical Turk, Prolific Academic, and Duke University's Behavioral Research Lab to participate in a general study about their views and attitudes regarding social topics. Participation was anonymous and voluntary, and participants provided their informed consent prior to their participation.
Ethics oversight	We have included the following statement in the manuscript:<br><br>The research project was approved by the IRB committee at Columbia University. Participants in all of the experimental studies completed an informed consent prior to commencing their participation.

Note that full information on the approval of the study protocol must also be provided in the manuscript.

Field-specific reporting

Please select the one below that is the best fit for your research. If you are not sure, read the appropriate sections before making your selection.

☐ Life sciences ☒ Behavioural & social sciences ☐ Ecological, evolutionary & environmental sciences

For a reference copy of the document with all sections, see nature.com/documents/nr-reporting-summary-flat.pdf

Behavioural & social sciences study design

All studies must disclose on these points even when the disclosure is negative.

Study description	All studies reported in the manuscript are quantitative, using correlation and/or experimental methods.
Research sample	Participants in Studies 2a, 2d, 4, 5A and 5B were recruited via Amazon's Mechanical Turk, a commonly used platform in the social sciences for recruitment of research participants. Participants in Studies 2b and 2c were recruited via Prolific Academic, an alternative research platform. Although the sample of participants skews a bit younger than the general population, previous research has shown that responses collected on this sample population are equivalent to responses collected on other samples, both online and in person. Participants in Study 3 were recruited by the Duke Behavioral Research lab. All demographic information, including age, gender, and racial breakdown, are provided in the manuscript. Finally, Study 1 analyzed responses from U.S. respondents across five different waves of the World Values Survey - a publicly available dataset available from https://www.worldvaluessurvey.org/ - and the Opportunity Insights dataset (including data from the 2000 U.S. census; https://rajchetty.com/wp-content/uploads/2021/04/land-of-opportunity.html-charsetutf-8).
Sampling strategy	Sample size for Studies 2-5 were determined in advance for each study, based on the observed effect in previous pilot studies and keeping in minds the availability of funds. The manuscript reports sensitivity analyses for all studies, computing the minimum effect size that can be detected with 80% power given each sample size.
Data collection	Data collection in all studies was conducted online. Participants were directed from Amazon Mechanical Turk, Prolific Academic, or Duke University's Research lab to an external survey hosted by Qualtrics XM - a research survey platform - and thus completed the study independently, without any researchers' presence. The fact that participants did not know in advance the research topic and that they were randomly assigned to condition minimizes concerns regarding self-selection bias. Participation in all studies was anonymous and voluntary.
Timing	All studies were collected between October 2021 and August 2023
Data exclusions	As noted in the manuscript, participants who failed a simple attention check described in the manuscript were excluded from analyses in Studies 2-5B:<br>Study 2a - 9 participants<br>Study 2b - 8 participants<br>Study 2c - 6 participants<br>Study 2d - 18 participants

Study 3 - 10 participants
Study 4 - 31 participants
Study 5A - 15 participants
Study 5B - 11 participants

Non-participation

All studies were conducted online and therefore there was no tracking of drop out' rate

Randomization

Participants in Studies 2a, 2b, 2c, 2d, 3, 4, 5a, and 5B were randomly assigned to experimental condition by the Qualtrics software. Study 1 is a correctional analysis of a publicly available archival dataset. As described in the manuscript, our analyses controlled for multiple potential covariates, including economic inequality, political leaning, racial composition, population size, income per capita, and racial segregation.

Reporting for specific materials, systems and methods

We require information from authors about some types of materials, experimental systems and methods used in many studies. Here, indicate whether each material, system or method listed is relevant to your study. If you are not sure if a list item applies to your research, read the appropriate section before selecting a response.

Materials & experimental systems

n/a	Involved in the study
☒	☐ Antibodies
☒	☐ Eukaryotic cell lines
☒	☐ Palaeontology and archaeology
☒	☐ Animals and other organisms
☒	☐ Clinical data
☒	☐ Dual use research of concern
☒	☐ Plants

Methods

n/a	Involved in the study
☒	☐ ChIP-seq
☒	☐ Flow cytometry
☒	☐ MRI-based neuroimaging

Plants

Seed stocks

n/a

Novel plant genotypes

n/a

Authentication

n/a
